# Pre-Analytical Considerations for Successful Next-Generation Sequencing (NGS): Challenges and Opportunities for Formalin-Fixed and Paraffin-Embedded Tumor Tissue (FFPE) Samples

**DOI:** 10.3390/ijms17091579

**Published:** 2016-09-20

**Authors:** Gladys Arreaza, Ping Qiu, Ling Pang, Andrew Albright, Lewis Z. Hong, Matthew J. Marton, Diane Levitan

**Affiliations:** 1 Translational Medicine, Merck Research Laboratories, Merck & Co., Inc., Kenilworth, NJ 07033, USA; gladys.arreaza@merck.com (G.A.); ling.pang@merck.com (L.P.); andrew_albright@merck.com (A.A.); matthew_marton@merck.com (M.J.M.); diane.levitan@merck.com (D.L.); 2 Translational Biomarkers, Merck Research Laboratories, Merck Sharp & Dohme, Singapore 609927, Singapore; zuocheng.lewis.hong@merck.com

**Keywords:** next-generation sequencing (NGS), formalin-fixed and paraffin-embedded tumor tissue (FFPE), pre-analytics, DNA extraction, DNA amplifiability

## Abstract

In cancer drug discovery, it is important to investigate the genetic determinants of response or resistance to cancer therapy as well as factors that contribute to adverse events in the course of clinical trials. Despite the emergence of new technologies and the ability to measure more diverse analytes (e.g., circulating tumor cell (CTC), circulating tumor DNA (ctDNA), etc.), tumor tissue is still the most common and reliable source for biomarker investigation. Because of its worldwide use and ability to preserve samples for many decades at ambient temperature, formalin-fixed, paraffin-embedded tumor tissue (FFPE) is likely to be the preferred choice for tissue preservation in clinical practice for the foreseeable future. Multiple analyses are routinely performed on the same FFPE samples (such as *Immunohistochemistry* (IHC), in situ hybridization, RNAseq, DNAseq, TILseq, Methyl-Seq, etc.). Thus, specimen prioritization and optimization of the isolation of analytes is critical to ensure successful completion of each assay. FFPE is notorious for producing suboptimal DNA quality and low DNA yield. However, commercial vendors tend to request higher DNA sample mass than what is actually required for downstream assays, which restricts the breadth of biomarker work that can be performed. We evaluated multiple genomics service laboratories to assess the current state of NGS pre-analytical processing of FFPE. Significant differences in pre-analytical capabilities were observed. Key aspects are highlighted and recommendations are made to improve the current practice in translational research.

## 1. Introduction

### 1.1. FFPE Remains the Standard of Practice

Formalin-fixed, paraffin-embedded tumor tissue (FFPE) and ultralow temperature frozen tissue (at −80 to −190 °C) are the most widely used methods to preserve nucleic acid, protein and histology for diagnostics and research purposes. Ultralow temperature frozen tissue is the current gold standard biospecimen for translational research. Fresh frozen tissue might be the preferred recommended source for certain next-generation sequencing (NGS) applications in which larger amplicons are required such as studying the tumor-infiltrating T cell immune repertoire in tumor tissues. However, ultralow temperature storage requires facility space and infrastructure such as electricity, expensive equipment and its maintenance and handling cost. FFPE is the ubiquitous room temperature clinical tissue biospecimen preservation method, even though formalin fixation leads to cross-linked and fragmented nucleic acids, denatured proteins and DNA modifications at a rate of one modification per 500 bases. Other ambient temperature preservation methods have limitations. For example, long-term ambient temperature storage desiccated chemical matrices are currently limited to nucleic acids only [1]. Formalin free fixatives show varying abilities to preserve nucleic acids. Cost also prohibits them from replacing FFPE specimens [1]. Thus, FFPE will remain the standard of practice for tissue preservation in the near term.

### 1.2. Factors Impacting the Quality of FFPE Samples

Sample handling in the operating room, formalin fixation, paraffin embedding and storage methods all impact sample quality. Ideally, samples should be fixed as soon as possible to reduce ischemic time. The length of formalin fixation is also critical to sample preservation. Under-fixation can lead to nucleic acid and protein degradation or a change in gene expression in deeper regions of the tissue specimen that have not been penetrated by formalin. Over-fixation can result in more extensive crosslinking, which makes extraction of usable nucleic acids and proteins more difficult. The types of DNA damage in formalin-fixed tissues include: (1) formaldehyde-induced crosslinks; (2) DNA fragmentation; (3) deamination of cytosine bases leading to C->T mutations; and (4) generation of abasic sites [2]. It is recommended to store FFPE blocks at lower temperatures (4 °C) instead of room temperature to prolong storage time [3].

### 1.3. Critical Considerations for Retrieval of DNA from FFPE Samples

There are several major difficulties when purifying DNA from FFPE samples due to biomolecule crosslinking, nucleic acid fragmentation and low yield. Therefore, the purification procedure used needs to be highly efficient, enabling the recovery of as much usable analyte as possible. There are many ready-to-use commercially available kits dedicated for DNA extraction from FFPE tissues. The majority of them are column-based; some use magnetic bead-based DNA reversible binding technology. Janecka et al. [4] compared the effectiveness of eight commercially available kits for DNA extraction based on 10 FFPE tissues. The results showed that the kits differ significantly in terms of yield, purity, and quality of the extracted DNA. They also demonstrated that overnight Proteinase K digestion of samples usually improves DNA yield and/or purity. For precious or limited material, double elution is recommended for obtaining up to 42% higher recovery of DNA. Heydt et al. [5] evaluated five automated FFPE DNA extraction systems as well as five DNA quantification systems using the three most common techniques, UV spectrophotometry, fluorescent dye-based quantification and quantitative PCR, on 26 FFPE tissue samples. The authors concluded that it is particularly important to choose the most reliable and consistent DNA extraction system, especially when using small biopsies and low elution volumes, and that all commonly used DNA quantification techniques are suitable for downstream applications such as massively parallel sequencing.

Next-generation sequencing (NGS) is a promising technology being used in the clinic to direct patient treatment. NGS-based assays are now transitioning into molecular pathology laboratories [6,7,8,9]. Gene panel-based NGS assays are regularly used for cancer subtype diagnosis using either fresh frozen or FFPE tissue [10]. Several studies have demonstrated that higher somatic non-synonymous mutational burden assessed by whole exome sequencing (WES) is associated with the clinical efficacy of anti-PD-1/anti-PD-L1 therapy [11,12,13]. Two of the most significant steps that contribute to the variability of the NGS assay are NGS pre-analytics and the bioinformatics data analysis pipeline. We have previously reported data interoperability of whole exome sequencing (WES)-based mutational burden estimates from different commercial laboratories and highlighted that mutational burden results from different labs should not be compared directly [14]; however, the discrepancy between labs can be mitigated by unified data analysis. Here, in this report we examine one step earlier in the process to investigate and compare the current state of FFPE NGS pre-analytics from commercial labs, particularly focused on the quantity and quality of DNA extracted from FFPE samples and the common DNA quality control (QC) practices currently being applied.

## 2. Current State of FFPE NGS Pre-Analytics in Commercial Labs and Recommendations for Improvement

In practice, when choosing a central laboratory or contract research organization (CRO) for NGS or DNA/RNA extraction, it is very critical to benchmark the lab performance using commercial reference samples, such as HorizonDx FFPE cell line curls, or FFPE slides that have been extracted and benchmarked previously. Twenty FFPE blocks (colorectal cancer, renal cell carcinoma, breast cancer, lung cancer) of different ages (ranging from one to five years) were sectioned to 5 µm slides (50 slides for each block). Five slides from each subject were sent to four commercial labs for DNA extraction. Slides were arranged in a way to minimize bias. For example, slides 1, 11, 21, 31, 41 were sent to Lab A, slides 2, 12, 22, 32, 42 were sent to Lab B, and so on. Each lab quantified DNA (both yield and fraction of amplifiable DNA) using its preferred method. Each lab sent the remaining DNA back to us for standardized testing of DNA quantitation and DNA amplifiability. 

### 2.1. Substantial Variability in Commercial Laboratory Performance on DNA Extraction

**Figure 1:**
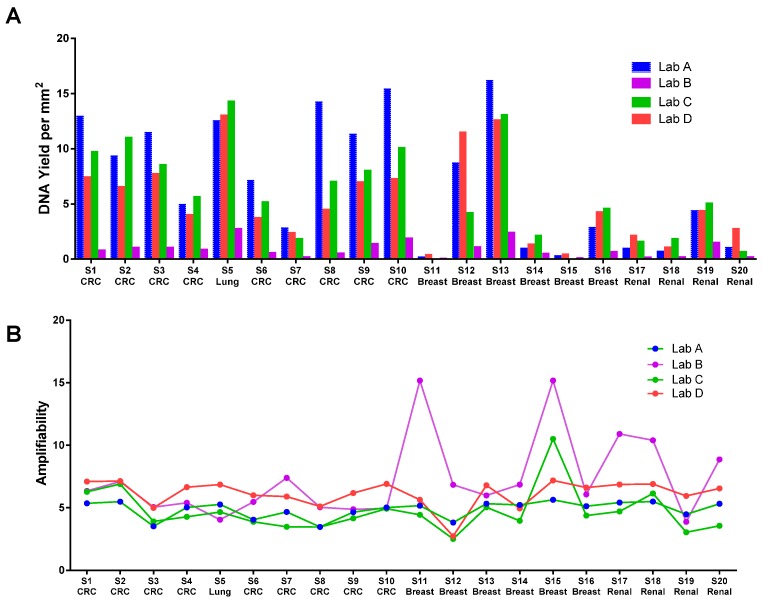
(**A**) DNA yield (ng/mm^2^ tissue) from different labs. QIAamp FFPE kit (QIAGEN Cat# 56404, Hilden, Germany) was used by Labs A, C, D. RecoverAll (ThermoFisher Cat# AM1975, Waltham, MA, USA) was used by Lab B; (**B**) DNA amplifiability from different labs. Amplifiability values have been converted to the same log 2 scale (*C*_t_ value). Values across labs are not comparable due to lack of common reference DNA. Values from the same lab correlate with the quality of FFPE.

The DNA yield from commercial labs from the same FFPE blocks varied significantly as measured by Qubit. For example, DNA yield from Lab B was generally five to 10 times lower than Lab A (Figure 1A). Substantial inter-lab variation in DNA yield was observed when the same FFPE DNA kit was used for DNA extraction (Figure 1A), and substantial intra-lab variation was observed when the same lab used different kits (two kits were used by Lab C, data not shown). We quantified all DNA using a Nanodrop spectrophotometer and a Qubit fluorometer. Qubit DNA quantification by our lab (Qubit High Sensitivity Assay) is in close agreement (*R*^2^ range from 0.79 to 0.97) with the Qubit readings from commercial labs which indicates that the yield discrepancy is not caused by DNA quantification. Although the readings from the Qubit assay and Nanodrop are strongly correlated, the Nanodrop readings are generally two to 10 times higher than the Qubit readings; this is likely caused by residual RNA, denatured single-stranded DNA (ssDNA) during the DNA extraction and other contaminants in the sample. Results from different FFPE DNA quantification methods are not comparable. We strongly recommend that a consistent method is used in each lab. We have seen very good correlation between the Qubit result and droplet digital PCR (ddPCR) quantification of reference DNA (data not shown). In addition, our result demonstrated that the Qubit result showed the best consistency in quantifying DNA extracted from same set of FFPEs by different labs. Therefore, Qubit is a preferred choice in our opinion for FFPE DNA quantification.

### 2.2. Lack of Standardization in DNA Input Requirement for NGS

Clinical tissue samples are often minute in quantity, as needle biopsies become common [15,16]. NGS assays such as WES generally require high DNA input amounts and it is critical to ensure optimal DNA input to warrant a satisfactory result. Insufficient DNA input often leads to library preparation failure or a high degree of sequencing artifacts [17,18,19], while high input can lead to inefficient use of limited sample.

The requirement for DNA input mass will vary depending on the type of NGS application, DNA quality, required detection limit, choice of library preparation protocol, etc. Generally, the minimum DNA input requirement depends on the performance requirements and the efficiency of the method employed (e.g., adapter ligation efficiency, capture efficiency). Rykalina et al. [20] achieved successful WES library preparation starting from as low as 10 ng DNA. Chung et al. [21] compared various commercial kits, optimized the library preparation protocol and evaluated the performance of the optimized protocol using different DNA inputs ranging from 6.25 ng to 200 ng. They found that 6.25 ng was sufficient to detect >99% of base substitutions with minor allele frequency (MAF) >15%. It is important to note that these studies all used intact, not-FFPE-derived, genomic DNA. Therefore, the DNA input requirement for FFPE samples is likely to be significantly higher.

Different commercial labs in our previous and current study have different DNA input requirements for each DNA-based assay. For example, the FFPE DNA requirement ranges from 50 ng to 1 µg for WES performed at different labs. The high-end DNA input requirement is normally based on a conservative estimate regardless of the DNA quality; however, the labs normally use the entire DNA provided, which often leads to a waste of precious DNA that could have been allocated for other assays. In some cases, the assay still fails due to severely low DNA quality, which indirectly results from a low quantity of usable DNA. Several labs have adopted a strategy to estimate amplifiable DNA in FFPE samples, although not all labs are implementing this approach as routine practice, and currently there is no standard in amplifiability testing. In cases where the DNA amount is very limited and multiple assays are required, we recommend that a DNA amplifiability test be performed, as well as a feasibility assessment of NGS based on the available tissue and DNA quality. The appropriate amount of input DNA amount can then be allocated for the NGS-based DNA assay if determined to be practical.

### 2.3. Lack of Standard and Routine Implementation of Functional DNA QC Test in Commercial Labs

Different companies normally use different versions of PCR-based assays to evaluate FFPE DNA amplifiability; some are commercial kits and some are laboratory-developed tests. We found that most commercial labs involved in our previous study do not routinely perform amplifiability tests as the guide for NGS DNA input. The fraction of functional DNA from the same FFPE tissue block in the extracted DNA could be very different due to different wet lab processes by different labs and the matrix in the resulting extracted DNA. We requested each lab to quantify the amount of amplifiable template using their preferred methodology, which included ddPCR, the Illumina FFPE QC Kit and laboratory self-developed qPCR kits. Separately, we carried out an independent evaluation of DNA quality from each lab by running the same amplifiability assay on these samples.

**Figure 2:**
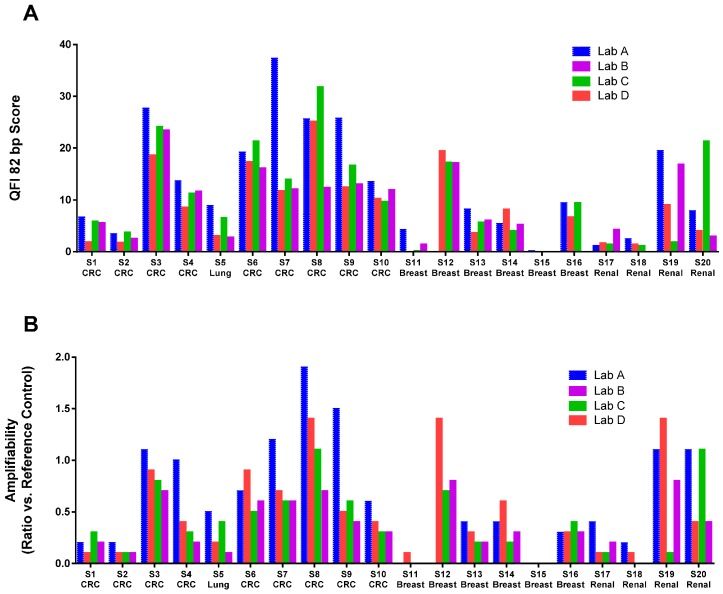
Amplifiability of DNA extracted by different labs measured by Asuragen Quantitative Functional Index (QFI) (**A**) and RNase P (**B**) assays. S12 from Lab A was run out and quality control (QC) data is not available. Missing data for S11 is due to poor FFPE block quality and high fat content of the breast tissue. CRC: colorectal cancer.

Amplifiability was reported by the labs in several different formats: percentage of amplifiable DNA, cycle threshold (*C*_t_) value, and Δ*C*_t_ value, which cannot be directly compared without a common reference. Figure 1B shows that the amplifiability test results from all labs after conversion of the data into the same number scale (log conversion for the percentage and subtraction of the common reference *C*_t_ to derive the Δ*C*_t_). As demonstrated, all methods reported a very similar relative quality of the FFPE samples. It should be noted that the absolute values are not compatible and should not be combined for sample quality comparison across labs due to different data reporting formats. To compare the quality of DNA extracted by different labs, we independently performed the Asuragen QuantideX qPCR DNA QC Assay (Asuragen Cat#49539, Austin, TX, USA) and another qPCR-based RNase P test on all samples extracted from all four labs (Figure 2). QuantideX qPCR DNA QC Assay is a multiplex qPCR assay with one channel that detects an 82-bp amplicon from the *TBP* gene, which assesses sample DNA quality and quantity (QFI-82). The RNase P assay is an in-house-developed qPCR-based test interrogating a 87 bp amplicon that maps within the single exon *RPPH1* gene (Applied Biosystems Cat#4403326, Foster City, CA, USA). Amplification is compared to a reference DNA (K562, Promega Cat#DD2011, Madison, WI, USA). We found significant inter-lab and inter-kit differences in the amount of amplifiable DNA from the same set of FFPE blocks. For example, sample S7 was extracted by Labs A, C and D using the same QIAamp FFPE kit, but we observed a large difference in DNA amplifiability. It was also interesting to note that DNA amplifiability measured by different tests could vary widely for the same DNA sample. For example, DNA extracted from S20 by Lab A demonstrated quite different amplifiability when using the Asuragen test versus the RNase P test. Therefore, we recommend that each laboratory qualifies a DNA amplification test for their workflow and uses it consistently to evaluate FFPE sample quality. With a consistent DNA extraction method and a consistent DNA amplifiability test, the minimum NGS DNA input can be practically established for each FFPE sample.

### 2.4. Adjust NGS Input Based on DNA Amplifiability Test

WES is still an expensive assay. In many cases, the experiment can be stopped prior to sequencing to avoid the more expensive sequencing step by critically monitoring pre-analytical QC steps. Given the lack of guidelines for the quality control of NGS pre-analytical steps and the lack of reference material, each lab should consider using a standardized DNA extraction kit and a DNA amplifiability test to ensure reproducible QC results. In addition to DNA QC metrics, before-capture and post-capture QC data of library preparation should be monitored and analyzed together with the final WES QC data (on target rate, average unique coverage and the uniformity of coverage, etc.) to guide the optimization of the NGS workflow to ensure the maximum success rate.

The optimal DNA input for NGS can be expressed as the minimum amount of DNA that enables the generation of a sufficiently complex library. In theory, 1 ng of intact DNA equates to 330 copies of the human genome, which should be sufficiently powered to detect mutations with 5% allele frequency and with 95% confidence if all copies are utilized in the assay. However, depending on the particular library prep protocol, only a certain percentage of the template can be recovered during the library prep and capture step and only a certain percentage of the starting DNA can be applied to the sequencing chip. Rykalina et al. (2014) and Chung et al. (2016) both demonstrated that satisfactory WES data can be achieved from ~10 ng of intact DNA. The minimum input FFPE DNA for WES can be estimated if the fraction of the functional DNA can be determined [20,21]. For instance, if, according to the amplifiability test, only 20% of the DNA is amplifiable in one FFPE DNA sample, the adjusted minimum starting DNA input should be five times higher. In our lab, by adjusting DNA input from 50 ng to 500 ng and 1000 ng for samples S1 and S2 (QFI-82 = ~ 5, 5% amplifiable DNA), we were able to make successful library preparations (pre and capture QC passed respective cutoffs) (Table 1).

## 3. Conclusions

It is essential to optimize pre-analytics in order to facilitate adequate NGS translational research. FFPE is anticipated to be the main tissue preservation choice for some time until another room temperature tissue preservation method becomes practical in clinical practice. Variability in FFPE preparation steps is hard to control across different clinical sites. It is best to require that the biospecimen preparation process be clearly documented by clinical sites. In addition to FFPE quality, different DNA extraction kits, labs/operators, and DNA amplifiability tests all contribute independently to the variability of yield and quality of extracted DNA which makes it difficult to evaluate each factor separately. Each lab should evaluate different DNA extraction and amplifiability tests independently and choose the best methods for the lab workflow using reference material. DNA input guidelines from each vendor should be looked at critically. The DNA input for an NGS assay should be determined by the amount of amplifiable DNA, not the absolute DNA amount. In practice, the CRO or testing lab should be evaluated holistically and followed with continuous improvement of each key component, ultimately correlating each step in the process to successful WES of the samples.

## Figures and Tables

**Table 1:** Whole exome sequencing (WES) library prep pre-capture and post-capture quality control (QC) data on samples with default 50 ng input and amplifiability adjusted input. Successful pre-capture QC cutoff = 700 ng and post-capture cutoff = 10 nM. The Agilent SureSelect Clinical Research Exome was used in this study.

Sample	FFPE Quality (QFI-82)	DNA Input		PreCapture PCR Yield (ng)		Post Capture PCR: Library Conc (nM)	
				Cutoff = 700 ng		Cutoff = 10 nM	
		Default (ng)	QFI Adjusted (ng)	50 ng Input	QFI Adjusted Input	50 ng Input	QFI Adjusted Input
S1	Low (6.0)	50	500	392.1	1662	6.4	13
S2	Low (3.4)	50	1000	424.5	1200	3.2	9.9
S5	Medium (8.9)	50	250	732	1911	14.9	19.5
S10	Medium (13.5)	50	250	1014	1911	10	22.5
S6	High (15.0)	50	100	1962	1974	18.5	14.9
S19	High (23.1)	50	100	1389	2094	12.5	10

FFPE: formalin-fixed, paraffin-embedded tumor tissue; QFI: Quantitative Functional Index.
